# Population dynamics and demographic history of Eurasian collared lemmings

**DOI:** 10.1186/s12862-022-02081-y

**Published:** 2022-11-03

**Authors:** Edana Lord, Aurelio Marangoni, Mateusz Baca, Danijela Popović, Anna V. Goropashnaya, John R. Stewart, Monika V. Knul, Pierre Noiret, Mietje Germonpré, Elodie-Laure Jimenez, Natalia I. Abramson, Sergey Vartanyan, Stefan Prost, Nickolay G. Smirnov, Elena A. Kuzmina, Remi-André Olsen, Vadim B. Fedorov, Love Dalén

**Affiliations:** 1 Centre for Palaeogenetics, Svante Arrhenius Väg 20C, 10691 Stockholm, Sweden; 2 Department of Zoology, Stockholm University, 10691 Stockholm, Sweden; 3 Department of Bioinformatics and Genetics, Swedish Museum of Natural History, Box 50007, 10405 Stockholm, Sweden; 4 Centre of New Technologies, University of Warsaw, S. Banacha 2C, 02-097 Warsaw, Poland; 5 Institute of Arctic Biology, University of Alaska Fairbanks, Fairbanks, AK 99775-7000 USA; 6 Faculty of Science and Technology, Bournemouth University, Talbot Campus, Fern Barrow, Poole, BH12 5BB Dorset UK; 7 Department of Archaeology, Anthropology and Geography, University of Winchester, Winchester, SO22 4NR UK; 8 Service de Préhistoire, Université de Liège, Place du 20 Août 7, 4000 Liège, Belgium; 9 OD Earth and History of Life, Royal Belgian Institute of Natural Sciences, Vautierstraat 29, Brussels, Belgium; 10 School of Geosciences, University of Aberdeen, Aberdeen, Scotland; 11 Department of Molecular Systematics, Zoological Institute RAS, St Petersburg, Russia; 12 Far East Branch, N.A. Shilo North-East Interdisciplinary Scientific Research Institute Russian Academy of Sciences (NEISRI FEB RAS), 685000 Magadan, Russia; 13 Central Research Laboratories, Natural History Museum Vienna, 1010 Vienna, Austria; 14 Department of Cognitive Biology, University of Vienna, 1090 Vienna, Austria; 15 Konrad Lorenz Institute of Ethology, 1160 Vienna, Austria; 16 South African National Biodiversity Institute, National Zoological Garden, Pretoria, South Africa; 17 Institute of Plant and Animal Ecology UB RAS, Russian Academy of Sciences, 202 8 Marta Street, 620144 Ekaterinburg, Russia; 18 Science for Life Laboratory (SciLifeLab), Dept of Biochemistry and Biophysics, Stockholm University, Stockholm, Sweden

**Keywords:** Collared lemming, Palaeogenomics, Demographic history, Population structure

## Abstract

**Background:**

Ancient DNA studies suggest that Late Pleistocene climatic changes had a significant effect on population dynamics in Arctic species. The Eurasian collared lemming (*Dicrostonyx torquatus*) is a keystone species in the Arctic ecosystem. Earlier studies have indicated that past climatic fluctuations were important drivers of past population dynamics in this species.

**Results:**

Here, we analysed 59 ancient and 54 modern mitogenomes from across Eurasia, along with one modern nuclear genome. Our results suggest population growth and genetic diversification during the early Late Pleistocene, implying that collared lemmings may have experienced a genetic bottleneck during the warm Eemian interglacial. Furthermore, we find multiple temporally structured mitogenome clades during the Late Pleistocene, consistent with earlier results suggesting a dynamic late glacial population history. Finally, we identify a population in northeastern Siberia that maintained genetic diversity and a constant population size at the end of the Pleistocene, suggesting suitable conditions for collared lemmings in this region during the increasing temperatures associated with the onset of the Holocene.

**Conclusions:**

This study highlights an influence of past warming, in particular the Eemian interglacial, on the evolutionary history of the collared lemming, along with spatiotemporal population structuring throughout the Late Pleistocene.

**Supplementary Information:**

The online version contains supplementary material available at 10.1186/s12862-022-02081-y.

## Background

The climatic fluctuations of the Late Pleistocene were an important driver of species divergence, genetic diversity, population structure, and demography [1–4]. In the Arctic, cold-adapted species expanded their ranges during glacial cycles, and became restricted to refugia during warm interglacials [5]. These changes in global distribution likely had impacts on their evolutionary history [6]. However, for species that were preyed upon by humans (i.e. the megafauna), it can be difficult to discern between the effects of climate and humans on population structure and demography [4, 7]. Small mammals on the other hand, were in all likelihood not preyed upon extensively by humans and can thus offer a unique opportunity to provide insights into the effects of the climate fluctuations on genetic diversity and demography.

Collared lemmings (*Dicrostonyx spp.*) are small cold-adapted rodents present in tundra environments across the Holarctic [8]. *D. torquatus* is present in Eurasia, with the current distribution encompassing the Arctic regions from western Russia to northeastern Siberia [9]. However, the fossil record indicates that *D. torquatus* had a much larger geographical distribution during the Late Pleistocene, encompassing central and western Europe [10, 11]. Additionally, a number of *Dicrostonyx* morphotypes have been described in the Palaearctic during the Pleistocene, some of which may be chronospecies (i.e. evolved sequentially), and have been used as biostratigraphic markers [12]. *D. renidens* (Early Pleistocene), *D. simplicior* (Middle Pleistocene), and *D. gulielmi* (Late Pleistocene) are suggested to have replaced one another through time. *D. gulielmi*, however, is likely a variant morphotype within *D. torquatus* that predominates during the Late Pleistocene [12, 13].

Previous studies on short mitochondrial DNA sequences have identified five lineages present in *D. torquatus*, with only one of these lineages persisting today [1, 2, 13]. Demographic inferences have suggested serial extinction and replacement of these lineages, and that these may have been linked to climate fluctuations within the Late Pleistocene, notably Greenland interstadials 5 and 2. Recent evidence suggests that there is geographic population substructure within the modern lineage, with populations on either side of the Kolyma river having distinct population histories [14]. The timing of when this modern lineage arose is currently unclear, however, with some studies suggesting that this took place at the beginning of the last glacial period (104 thousand years before present [ka BP]) [14], but others during or after the Last Glacial Maximum (LGM, 28.6–22.5 ka BP, [15]) [2].

Ancient DNA studies on remains of small to medium sized mammals from non-permafrost sites have shown differing levels of DNA preservation [1, 2, 16–18], and most studies have focused only on short regions of mitochondrial DNA amplifiable by polymerase chain reaction (PCR). However, next generation sequencing is well-suited to the fragmented nature of ancient DNA and can be used to reconstruct complete mitogenomes and evaluate levels of endogenous DNA in ancient remains (e.g., [19]). Complete mitogenomes, in combination with dated remains, can be used to better estimate the substitution rate and divergence times. Here we aimed to recover complete mitogenomes from ancient collared lemmings across Europe and Siberia in order to explore Late Pleistocene population dynamics and investigate the discrepancy in timings of the emergence of *D. torquatus* lineages. Additionally, we aimed to reconstruct the demographic history of *D. torquatus* using a modern nuclear genome to further investigate the effects of climate on the evolutionary history of collared lemmings.

## Results

### De novo assembly, nuclear genome and ancient mitogenomes

A de novo reference assembly was constructed for the Eurasian collared lemming, *Dicrostonyx torquatus*, from modern tissue*.* The final draft assembly (allpaths) gave an assembly size of 2.5 Gb, comprising 31,150 scaffolds and a scaffold N50 of 6.0 Mb. We aligned the short reads generated for the assembly against the de novo reference, producing a nuclear genome with 36.9 × coverage (Additional file 1: Table S1).

**Fig. 1:**
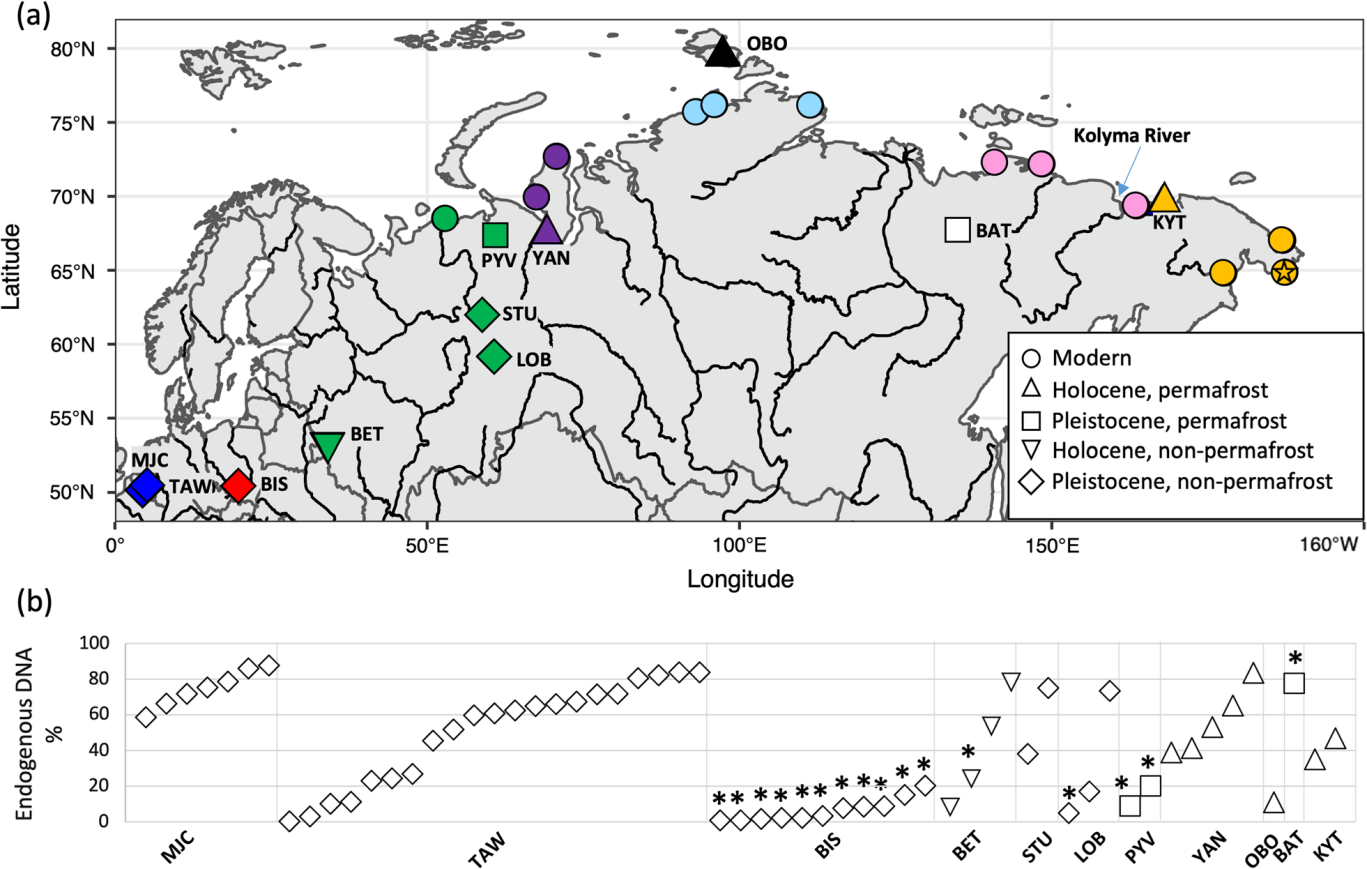
Map of sample locations and endogenous DNA content in sites. **a** Location of samples in the present study and modern samples from Fedorov et al*.* [14], coordinates can be found in Additional file 1: Tables S2 and S3. The locations of the modern samples represent the current distribution of *D. torquatus*. Star depicts the location of the modern genome sample. Major rivers displayed in black, with the Kolyma River indicated with an arrow. Site abbreviations are as follows: MJC, Marie-Jeanne Cave; TAW, Trou Al’Wesse; BIS, Bisnik Cave; BET, Betovo; STU, Studennaya; PYV, Pymva Shor; YAN, Yanagana Pe-4; OBO, Ostrov Bolshevik; BAT, Batagaika Crater; KYT, Kyttyk Peninsula. Samples are coloured by geographic location used to group sites in the phylogenetic analyses: western Europe, blue; eastern Europe, red; western Russia, green; western Siberia (east of the Ural mountains), purple; central Siberia (Taimyr), light blue; central Siberia (Yana-Kolyma), light pink; central Siberia (Ostrov Bolshevik), black; eastern Siberia, orange; Batagaika, white. Map was created in R v3.6.1. **b** Endogenous DNA content of ancient samples that were shotgun sequenced (n = 59), with site abbreviations following above. Asterisks indicate samples that did not undergo bleach and predigestion treatment

Using ancient DNA extraction methods and shotgun sequencing, we recovered 59 complete mitogenomes from ancient collared lemmings across Eurasia, with the average coverage ranging from 3–43 × (Fig. 1a, Additional file 1: Table S2). Several samples from both permafrost and non-permafrost localities had very high endogenous DNA content (up to 83.5% and 87.6%, respectively) (Fig. 1b, Additional file 1: Table S2). In particular, we observed high levels of endogenous DNA in samples from Marie-Jeanne Cave (48–24 ka BP) in Belgium (58.6–87.6%). Samples from Trou Al’Wesse, also in Belgium had lower endogenous DNA content overall in the older layer (layer 15, ~ 43–36 ka BP; endogenous 3–26%), with similar exceptional preservation to Marie-Jeanne Cave in the younger layer (layer 12, ~ 31–17 ka BP; endogenous 23–83%). We found variable endogenous DNA contents (4.9–80.5%) in the non-permafrost samples from Russia, with some well-preserved samples. Among the permafrost-preserved specimens, all samples had > 8% endogenous DNA, but it is worth noting that the majority of these samples all date to < 2 ka BP, with the exception of the Batagaika sample that had an infinite radiocarbon date (> 50 ka BP), and two samples from Pymva Shor dating to ~ 15 ka BP.

### Diversification of collared lemming lineages

**Fig. 2:**
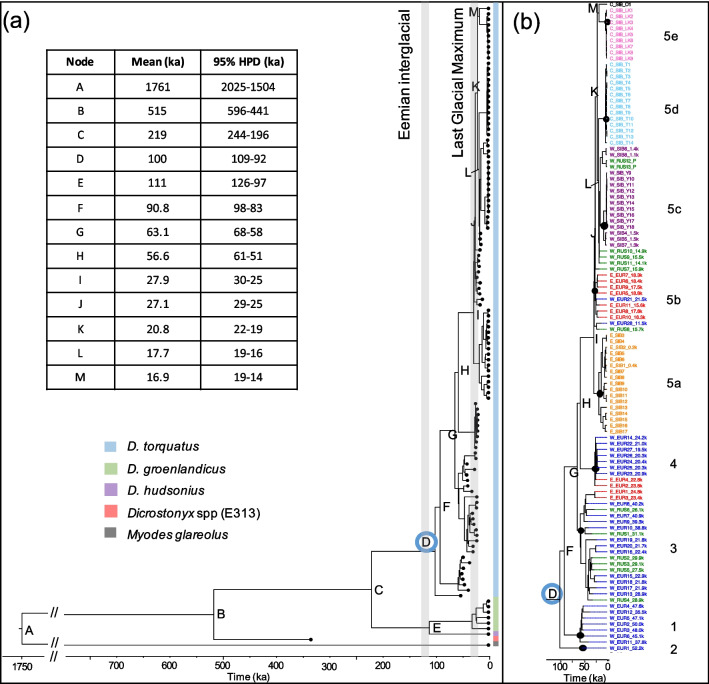
Mitochondrial phylogeny of collared lemmings (*Dicrostonyx spp.)* using BEAST v1.10.4. **a** Whole dataset, including *Myodes glareolus* as an outgroup. Major nodes are labelled and divergence times are listed in the table with mean age and 95% highest posterior density (HPD) given in thousands of years (ka). Blue open circle represents the most recent common ancestor of *D. torquatus*. Posterior support (not shown) for all major nodes was 1. Grey vertical bars show the Eemian interglacial (130–115 ka) and Last Glacial Maximum (28.6–22.5 ka). **b** *Dicrostonyx torquatus* phylogeny showing the distinct clades, identified by the black closed circles. Samples are coloured by geographic location: western Europe, blue; eastern Europe, red; western Russia, green; western Siberia (east of the Ural mountains), purple; central Siberia (Taimyr), light blue; central Siberia (Yana-Kolyma), light pink; central Siberia (Ostrov Bolshevik), black; eastern Siberia, orange. Time in both figures is given in thousands of years (ka)

The Bayesian phylogenetic tree showed that the mitogenomes of all extant *Dicrostonyx* species share a common ancestor ~ 219 ka BP (Node C, Fig. 2; Additional file 1: Table S4), coalescing during Marine Isotope Stage (MIS) 7 (243–191 ka BP). However, one ancient sample (E313), recovered from the Batagaika Crater, fell outside the diversity of all other samples, and diverged from all extant *Dicrostonyx* mitogenomes ~ 515 ka BP. This specimen had an infinite radiocarbon date, and we therefore used the molecular clock to estimate the age of this sample to ~ 333 ka BP (95% Highest posterior density [HPD]: 451–220 ka BP) (Additional file 1: Table S5), which fits within the date range for the Batagaika Crater (650–0 ka BP) [20]. We find that both the Eurasian (*D. torquatus*; node D, ~ 100 ka BP) and North American (*D. hudsonius* and *D. groenlandicus*; node E, ~ 111 ka BP) collared lemming lineages diversified after the Eemian interglacial (MIS 5e, 130–115 ka BP), which indicates that both these lineages may have gone through bottlenecks during this time period. Moreover, our results show that the clade encompassing all modern *D. torquatus* had a most recent common ancestor (mrca) at ~ 27 ka BP (node I, Fig. 2a).

### Phylogenetic structure and genetic diversity of *D. torquatus*

Within *D. torquatus*, we confirmed the existence of the five previously identified mitochondrial clades, each with high posterior probabilities (clades 1–5, Fig. 2b), and with clade 5 encompassing all modern samples. Interestingly, sample W_EUR1 is the earliest branching clade within *D. torquatus* (clade 2, Fig. 2b), with high posterior probability (PP = 1). This sample was previously classified as EA2, the second of the five Eurasian clades identified in Palkopoulou et al. [2]. We find little geographic structure within clades 1–4, although we note that our data does not consist of samples from across the entire Late Pleistocene geographic range for each of these clades. Our data suggests that clades 1, 2, 3, and 4 overlapped temporally, but each of these clades had become extinct towards the end of the Last Glacial Maximum. Clade 5 comprises Late Pleistocene samples from Europe and western Russia, as well as Holocene and modern samples from across the species’ current range. Within this clade, we observe the substructure previously identified in modern *D. torquatus* [14], but with the addition of a distinct subclade (5b) comprising late and post-LGM samples from Belgium and Poland.

Using the nuclear genome, we estimated genome-wide heterozygosity of the modern specimen from northeastern Siberia to be 5.97 heterozygous sites per 1000 bp. We did not identify any runs of homozygosity within the genome (> 100 kb), despite investigating a range of parameters.

### Demographic history of *D. torquatus*

**Fig. 3:**
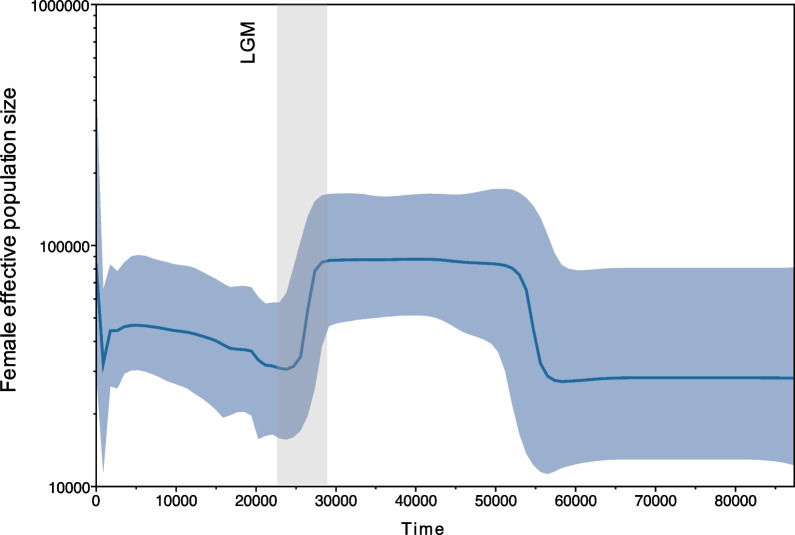
Mitochondrial demographic reconstruction of *Dicrostonyx torquatus* over the past 80 ka BP, constructed using BEAST 1.10.4. Female effective population size is given in a log scale. Light blue shows the 95% confidence interval. The grey bar represents the Last Glacial Maximum (LGM, 28.6–22.5 ka BP)

The demographic reconstruction of *D. torquatus* based on the mitogenome data suggested that the female effective population size (*N*_*ef*_) increased between ~ 57–49 ka BP and then remained stable (at *N*_*ef*_ =  ~ 90,000) for ~ 21 ka (Fig. 3). *D. torquatus* then went through a decline from ~ 28–21 ka BP that appears to coincide with the onset of the LGM. Following the decline, there was a gradual increase in *N*_*ef*_ from ~ 22–5 ka BP, until a brief bottleneck in the late Holocene. However, we note there are large confidence intervals in the demographic analysis, especially surrounding the bottleneck stages. We further tested the effect of Eemian interglacial and LGM bottlenecks using Approximate Bayesian Computations (ABC) of simulated data, accounting for the population structure we observe in the dataset. The ABC analysis indicates support for a model with a bottleneck during both the Eemian interglacial and the LGM (Additional file 1: Table S6).

**Fig. 4:**
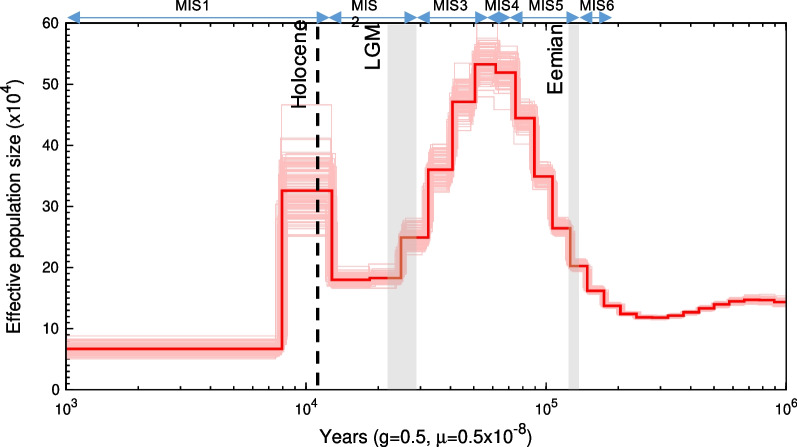
Demographic reconstruction of effective population size of *Dicrostonyx torquatus* using PSMC. The PSMC curve is scaled with a generation time (g) of two generations per year [23] and a mutation rate (μ) of 5.4 × 10^–9^ substitutions per site per generation [22]. The x axis shows time on a log scale. The dashed line represents the Pleistocene-Holocene boundary (~ 11.7 ka BP). Grey bars indicate the Last Glacial Maximum (LGM, 28.6–22.4 ka BP) and the Eemian interglacial (130–115 ka BP). Blue arrows and MIS labels designate Marine Isotope Stages 1 to 6. Light red lines represent 100 bootstrap replicates

To further explore the demographic history of *D. torquatus*, we undertook a PSMC [21] analysis of the modern nuclear genome from an individual from northeastern Siberia (Western Beringia) (Fig. 4), belonging to the mitochondrial haplogroup 5a. As the mutation rate is unknown for collared lemmings, we used three rates obtained from the mouse (*Mus musculus*) to scale our analyses [22] (see Materials and Methods). When using the average mutation rate (5.4 × 10^–9^ substitutions/site/generation), the effective population size (*N*_*e*_) increased from ~ 220 ka BP, reaching a peak at 55–50 ka BP during the beginning of MIS 3 (57–29 ka BP). From the peak, *N*_*e*_ declined until ~ 10.2 ka BP, where there was an increase in *N*_*e*_. The effective population size then decreased and subsequently remained constant from ~ 7.9 ka BP (at *N*_*e*_ =  ~ 70,000) until present day. Additional analyses with a range of mutation rates produced similar results, with the curve shifting on the time axis (i.e. faster mutation rate shifts the curve to the left, Additional file 2: Fig S1).

## Discussion

### Impact of climate on *Dicrostonyx*

Our mitogenome results suggest that the diversity within each collared lemming species evolved after the Eemian interglacial (130–115 ka BP) (Fig. 2a), and indicate that the mitogenomes of the North American species, *D. hudsonius* and *D. groenlandicus,* diverged from a common ancestor around this time. This implies that collared lemmings in both Eurasia and North America may have undergone bottlenecks during the Eemian interglacial as indicated by our ABC modelling, although we were not able to formally test this for the North American samples. During the Eemian, temperatures were ~ 2–5 degrees higher than current levels [24]. Cold-adapted species such as *Dicrostonyx* would likely have been contracted to refugia in northern Siberia and North America, with restricted gene flow between populations [25]. This probably led to genetic bottlenecks and loss of a substantial amount of genetic diversity. Mitochondrial divergence resulting from a bottleneck during the Eemian has been hypothesised for other cold-adapted species, including the woolly mammoth [26] and the woolly rhinoceros [27]. This implies that the Eemian interglacial likely had a significant impact on the evolutionary history of cold-adapted species across the Arctic. Future genomic studies utilising samples before and after the Eemian will be crucial in further evaluating the influence of this period on collared lemmings and other Arctic fauna.

### Substitution rates and divergence estimates

Using the molecular clock, we estimated the mrca of North American and Eurasian *Dicrostonyx* mitogenomes to ~ 219 ka BP and the mrca of the modern *D. torquatus* clade (clade 5) to ~ 27 ka BP. These estimates are both more recent than suggested by Fedorov et al. [14] (~ 760 ka BP and ~ 104 ka BP, respectively). This is due to differences in estimating the substitution rate between the two studies. Fedorov et al. [14] used a fossil calibration based on the date for the first fossil appearance of *Dicrostonyx* species in North America (~ 760 ka BP, [28]), giving a substitution rate of 5.6 × 10^−8^ (95% HPD: 4.0 × 10^−8^ to 7.3 × 10^−8^) substitutions/site/year, whereas the present study estimated the rate using the radiocarbon age of samples, giving a rate of 2.07 × 10^−7^ substitutions/site/year (95% HPD: 1.8 × 10^−7^ to 2.6 × 10^−7^ substitutions/site/year). The use of such a fossil calibration assumes that there have been no extinction-replacement events and/or population mixing after the date of the first identified fossil. The present study and previous literature have identified multiple lineages of *Dicrostonyx* occurring across their range throughout the Late Pleistocene [1, 2]. Although secondary admixture across the Bering strait was not detected in allele frequencies of multiple nuclear RAD loci [14], recently Fedorov et al. [29] suggested that gene flow resulted in mitochondrial replacement in the North American species. Thus, we propose that the divergence between Eurasian and North American *Dicrostonyx* occurred much more recently than the first identified North American fossil of *Dicrostonyx*, and that the substitution rate is much faster than previously assumed.

We note that it is plausible that post-speciation gene flow may have occurred amongst past linages of *Dicrostonyx.* This could be further investigated by generating nuclear genomic data from *D. groenlandicus*, and the Batagaika lemming (E313, Fig. 2a), which we suggest pre-dates the divergence between the Eurasian and North American *Dicrostonyx*. These data would allow us to estimate allele sharing between these species, with the hypothesis that the Batagaika lemming is symmetrically related to both the Eurasian and North American collared lemmings if the divergence between these species occurred after the date of the Batagaika lemming.

### Genetic structure in Eurasian collared lemmings

Using complete mitogenomes, we confirmed the presence of five distinct clades in *D. torquatus* (1–5) that were identified in previous *cytochrome B* studies [1, 2]. However, in the present study clade 2 diverges first, compared to clade 1 in previous studies [1, 2], which we suggest may be due to better phylogenetic resolution provided by the complete mitogenome data. Additional sequencing of previously identified clade 2 samples will help to elucidate whether the sample in our dataset forms a clade with the other clade 2 samples, or represents a distinct lineage.

As we do not present new radiocarbon dates for *Dicrostonyx* here, we were not able to investigate the temporal boundaries of each of the clades present in the mitogenomes. However, in contrast to the previous studies, we see an overlap in the mrca of the extinct clades, in particular clades 1 and 3 (mrca: 61–52 and 60–53 ka BP, respectively). This indicates that the clades themselves did not originate as a consequence of founder effects during recolonisation. Based on this, we hypothesise that *D. torquatus* may have survived as refugial populations in Europe during the short warm interstadials of MIS 3 and 2 (57–14 ka BP), which is supported by the presence of fossil evidence of *Dicrostonyx* as far south as France during this period [30]. Our mitogenome data does suggest a replacement of the Late Pleistocene clades 1–4 with clade 5 in the western most extent of their range, likely during the LGM, in agreement with Palkopoulou et al. [2]. Future genetic studies combined with direct radiocarbon dating would help to refine the temporal estimates of the distinct clades, and whether their appearance and disappearance were associated with major climatic shifts.

Clade 5 consists of geographically separated subclades (Fig. 2b). The eastern Siberian clade (5a) diverged first, suggesting an origin of clade 5 in this area and subsequent spread of collared lemmings during the end of the Late Pleistocene. Our results show that in Europe, clade 5 disappeared around 15–11 ka BP, which was also observed in previous studies [1, 2], and consistent with the fossil record [10]. This suggests that there may have been a rapid range contraction during the abrupt warming of the Bølling-Allerød interstadial (14.7–12.8 ka BP) or the transition to the Holocene (11.7 ka BP), leading to the decline and subsequent disappearance of collared lemmings from western Europe. Molecular clock dating of four ancient samples from western Russia (W_RUS7, 9, 10, 11), which are basal to clade 5c (western Siberia, Fig. 2B), suggests that these date to ~ 15.9–14.1 ka BP (95% HPD: 19–11 ka BP, Additional file 1: Table S5). Thus, our data indicates an eastward contraction of the collared lemmings’ range throughout the Bølling-Allerød interstadial, although radiocarbon dated samples from western Russia would be needed to confirm the timing of this contraction.

### Demographic history of *Dicrostonyx*

Our mitochondrial demographic analyses suggest stability in *N*_*ef*_ throughout much of MIS 3 (57–29 ka BP), despite increased interstadial and stadial events during this time. Following this, we observe a bottleneck during the LGM, beginning ~ 28 ka BP. Our ABC analysis additionally supported a model involving a bottleneck during the LGM (Additional file 1: Table S6). This is surprising for a cold-adapted species, and inconsistent with the fossil record which indicates a range expansion during this period [10]. We suggest this may be caused by the population structure present in our data, as all clades were included in the demographic analyses. We note that this violates the assumption of a panmictic population in the Bayesian Skyline analysis and can lead to false suggestions of declines in population size [31]. Alternatively, this may be due to the disappearance of clades 1–4 in Europe coinciding with an expansion of, and/or being replaced by clade 5 from a divergent source population during this time.

Based on analyses of the nuclear genome, we infer that collared lemming effective population size increased through MIS 6 (191–130 ka BP) and the Last Glacial period in northeastern Siberia, reaching a peak during MIS 4–3, depending on which mutation rate is assumed (Fig. 4, Additional file 2: Fig S1). A second, shorter peak in *N*_*e*_ seems to have occurred ~ 10 ka BP, coinciding with the early Holocene. However, instead of reflecting demographic changes, an alternative explanation for these peaks is that the population structure (as observed in the mitogenome data) may have led to spurious increases in the PSMC-based inferences of effective population size. Recent evidence suggests that the PSMC method is unable to correctly determine population size changes in highly structured populations [21, 32, 33], and can lead to increased effective population size observed in the PSMC. Thus, given our observed structure in the mitogenome data, we consider it plausible that the peaks in the PSMC, particularly around the onset of the Holocene where the confidence intervals are wider, may be the result of population structure as collared lemmings were separated into polar refugial populations during warm periods, or alternatively, mixing of the populations following times of warming, such as after the Bølling-Allerød interstadial (14.7–12.8 ka BP). Moreover, secondary admixture between the lineages of *D. torquatus*, as identified by allele frequencies of nuclear loci between Taymyr and West Beringia [14], may be further confounding the PSMC, which could be tested further in future with multiple ancient genomes. Multiple ancient genomes would also allow for calibration of the mutation rate (e.g., [34]), which would narrow down the time intervals when potential increases and decreases in population size occurred.

Within clade 5, Fedorov et al. [14] identified a pattern consistent with differing demographic population histories on either side of the Kolyma River, with the northwestern populations undergoing bottlenecks during the Holocene and the northeastern Siberian (West Beringian) populations remaining constant. This is confirmed in our nuclear data, where we find a small but stable effective population size from ~ 8 ka BP and high genetic diversity in the nuclear genome of an individual from northeastern Siberia. In addition, our analysis of runs of homozygosity in this genome suggested a lack of inbreeding in the northeast Siberian population, implying a lack of severe bottlenecks in its recent history. Recent paleoecological evidence suggests that, in contrast to the rest of the Eurasian Arctic, only a minor northward advance in the position of the tree line was detected [35] and tundra landscape dominated throughout the Holocene warming events [36, 37] in the extreme northwestern Siberia. Interestingly, ancient DNA evidence suggests that several other species demonstrated high diversity or unique lineages in northeastern Siberia, including muskox, wolves and woolly rhinoceros, as well as humans [3, 27, 38, 39]. The different demographic histories in extant populations of collared lemmings may also indicate the existence of a cryptic barrier to gene flow in the region of the Kolyma river during warmer periods, in particular the Holocene. While no evidence for ongoing gene flow has been documented so far between populations either side of the Kolyma river [14], ancient whole genome data from each of these sublineages will be imperative in testing whether gene flow occurred across the species’ range during warmer periods.

### DNA preservation

Studying ancient lemmings is a powerful way to investigate the impact of past climate change, but to do so in the future will rely on recovering autosomal palaeogenomic data. To do this, it is important to obtain high enough endogenous DNA for genome sequencing. Fortunately, we found very high levels of endogenous DNA in our samples, with the majority over 10%. This is likely due to the addition of a bleach wash and a pre-digestion step prior to DNA extraction, which has been shown to increase recovery of endogenous DNA in Atlantic cod, horse and humans [40, 41]. In particular, we found endogenous DNA contents ranging from 58–87% in samples from Marie-Jeanne Cave in Belgium (50–24 ka BP), suggesting that even non-permafrost material up to the limits of radiocarbon dating can have exceptionally good DNA preservation. Furthermore, we were able to recover a complete mitogenome from Batagaika, which we estimated to date to ~ 333 ka BP, with an endogenous DNA content of 77.4%. This sample likely represents one of the oldest mummified specimens identified to date, and allows for exploring evolutionary dynamics of *Dicrostonyx* deeper into the past. Thus, our results suggest that there is good preservation of DNA in *Dicrostonyx* fossil remains, and that future whole genome studies from non-permafrost, as well as permafrost, small mammal remains are feasible.

## Conclusions

Our results suggest an influence of climate warming in the Eemian interglacial on the evolutionary history of *Dicrostonyx* species, with subsequent diversification within each of the three extant species occurring during the last glacial period. We confirm the presence of distinct mitochondrial lineages present in *D. torquatus* during the Late Pleistocene, with only one lineage persisting throughout the Holocene. Within the lineage present today, there is evidence for geographically distinct mitochondrial lineages, and the analysis of the modern genome suggests that the northeastern Siberia (West Beringia) population maintained genetic diversity and a constant population size. This is likely due to preferable conditions for collared lemmings in the easternmost part of the species' distribution during the late glacial phase and transition to the Holocene. Overall, this study suggests a considerable influence of climate on the evolutionary history of collared lemmings and given the well-preserved nature of many of the samples, highlights the utility and potential of small mammals in future palaeogenetic studies on Pleistocene population dynamics.

## Materials and methods

### Ancient sample collection, DNA extraction and sequencing

We collected a total of 127 ancient samples of Eurasian *Dicrostonyx sp.* from 13 archaeological and paleontological sites. The DNA extractions and all pre-PCR work on the samples were performed in dedicated ancient DNA laboratories at the Swedish Museum of Natural History (NRM) and the Centre for Palaeogenetics (CPG) in Stockholm (Sweden), and the Laboratory of Paleogenetics and Conservation Genetics, Centre of New Technologies University of Warsaw, in Warsaw (Poland). All the procedures were performed with sterilized equipment and to minimize the risk of contamination from exogenous sources and between the samples [42].

For 97 of the samples (EL and MAM labelled), a bleach washing and predigestion step was undertaken, modified from Boessenkool et al. [40] (see Additional file 2: Supplementary Information). DNA was then extracted using the Protocol C from Yang et al. [43], as modified by Ersmark et al. [44]. All E-labelled samples (n = 19) were previously extracted in Palkopoulou et al. [2] using the modified Protocol C extraction, but without the bleach wash and predigestion. Double stranded libraries were prepared from the EL, MAM, and E-labelled extracts following Meyer & Kircher [45] (see Additional file 2). Equimolar pooled libraries were sequenced on either a 2 × 50 bp setup on an Illumina SPrime or a 2 × 100 bp setup on the Illumina NovaSeq S4 at Science for Life Laboratories (Sci*Life*Lab), Stockholm.

The remaining 11 (L-labelled) samples were extracted previously, as described in Palkopoulou et al. [2], using a phenol–chloroform protocol [46]. Double stranded libraries were prepared following Meyer and Kircher [45] with modifications [47] (see Additional file 2). Hybridisation capture was performed following Horn [48], with baits generated from vole species (common vole (*Microtus arvalis*), field vole (*Microtus agrestis*), root vole (*Microtus oeconomus*), bank vole (*Clethrionomys glareolus*) and narrow-headed vole (*Lasiopodomys gregalis*)) following Maricic et al. [49]. Captured libraries were purified, pooled in equimolar ratios, and paired-end sequenced on an Illumina NextSeq platform (2 × 150 bp, mid-output kit). In order to estimate endogenous DNA content, uncaptured libraries were also shotgun sequenced on an Illumina Nextseq 550 in a 2 × 150 bp, mid-output, paired-end setup.

### De novo genome assembly

A tissue sample from a modern *Dicrostonyx torquatus* individual (UAM:84102, Mammal Collection, University of Alaska Museum of the North) was obtained for de novo genome assembly. DNA was extracted using a Kingfisher Robot (Thermo Fisher Scientific) using the blood and tissue extraction protocol. The DNA concentration was measured using a Qubit® 2.0 Fluorometer (Invitrogen, USA) and DNA quality was assessed using gel electrophoresis. Library preparation, Genome sequencing and assembly from the DNA extract was performed at Sci*Life*Lab, Stockholm. In brief, five libraries were prepared from the extracted DNA: TruSeq PCR-free (180 bp), TruSeq PCR-free (670 bp), and three Nextera mate-pair libraries (1 × 3 kb, 1 × 5-8 kb and 1 × 20 kb). Each of the libraries were sequenced on an Illumina HighSeq X lane in a 2 × 150 bp setup. Three genome assembly methods were tested: SOAPdenovo [50], allpaths [51] and abyss [52] and evaluated using BUSCO [53].

### Bioinformatics on re-sequenced data

Raw resequencing data was demultiplexed using bcl2Fastq v2.17.1 (Illumina Inc.), and then trimmed and mapped to the reference de novo genome using a development version of GenErode (https://github.com/NBISweden/GenErode, [54]), where adapters were removed using a modified version of SeqPrep v1.1 (https://github.com/jstjohn/SeqPrep) as per [34]. Endogenous DNA content for all 59 samples was estimated from the BAM files prior to duplicate removal. The trimmed and merged reads for the E-, EL- and MAM-labelled samples were also mapped to the mitochondrial genome (Genbank ID: KX066190) using settings specific for ancient DNA: bwa aln, with deactivated seeding (-l 16,500), allowing more substitutions (-n 0.01) and allowing up to two gaps (-o 2). Mitochondrial BAM files were filtered for mapping quality and duplicates were removed using a custom python script. Consensus FASTA files were generated using Geneious v7.0.336 [55], with the majority rule and 3X coverage required to call positions, and any ambiguous positions remaining were called as N. One sample (E313) only mapped well to the conserved regions of the reference mitogenome, thus we constructed the mitogenome using MITObim v1.9.1 [56], with the reference mitogenome as a seed. The resulting consensus sequence was checked manually in Geneious.

For L-labelled captured samples, raw Illumina reads were demultiplexed using bcl2Fastq v2.20 (Illumina Inc.). AdapterRemoval v2.2.3 [57] was used for adapter and quality trimming sequences and to collapse paired-end reads. Merged reads were mapped to the reference mitochondrial genome using the mem algorithm in bwa v0.7.17 [58]. Only reads longer than 30 bp and with mapping quality over 30 were retained and duplicates were removed using the SAMtools package v1.9 [59]. Consensus sequences were called using the BCFtools v1.9 package [59]. We called positions with minimum 3X coverage. Each alignment was then inspected manually in Tablet [60].

For the specimen used for the de novo genome assembly, we merged and mapped the reads generated from the 180 bp inserts against the consensus genome sequence using a development version of GenErode, where trimmomatic [61] was used to trim reads.

### Mitogenome analyses

After mapping to the mitochondrial genome, we retained samples with > 94% total coverage and > 3X average coverage of the mitogenome for downstream analyses (Additional file 1: Table S2). We created two datasets: (1) ‘*D. torquatus’* comprising our 59 ancient mitogenomes and 54 modern, previously published, *D. torquatus* mitogenomes (Genbank: KX066190, MN792933-83; [14, 62]); and (2) ‘*Dicrostonyx* + *Outgroup*’ encompassing the *D. torquatus* dataset plus six modern *D. groenlandicus* (Genbank: KX712239, MN792984-8; [62]), one *D. hudsonius* (Genbank: KX683880; [62]), a mitogenome generated from a specimen identified as *Dicrostonyx sp.* (E313) with an infinite radiocarbon date (OxA-29747, > 50,100 cal BP), and an outgroup (*Myodes glareolus*, KM892817) (Additional file 1: Table S7). Both datasets were aligned using MUSCLE [63], and the nucleotide substitution models were determined in jmodeltest2 [64] to be GTR with a gamma distribution and invariant sites for both datasets.

To estimate divergence in the *Dicrostonyx* species, a Bayesian phylogeny was constructed in BEAST v1.10.4 using dataset 2, with an uncorrelated relaxed lognormal clock and coalescent constant size tree model. Dates were estimated for three samples with unknown or infinite radiocarbon ages; for the samples with unknown age a uniform prior (lower = 0, upper = 50,000) was used and for the infinite age sample a log normal prior (mean = 12.3, sigma = 0.325) was used. Samples with radiocarbon or estimated archaeological ages were given a normal prior with 97.5% above the minimum date (Additional file 1: Table S5). Three independent trees were run for 100 M generations each and combined using LogCombiner, removing 30% burnin. All BEAST runs were checked for convergence (effective sample sizes for parameters above 200) in Tracer. TreeAnnotator was used to generate a consensus tree, with 30% burnin removed. The Bayesian trees were visualised using Figtree v.3.4.3 [65].

Demographic reconstruction of *D. torquatus* (dataset 1) was undertaken using BEAST v1.10.4 [66] testing three different coalescent tree models: constant size, Bayesian skyline and Bayesian skyride. Trees were run for 100 M generations, with sampling every 1000 generations. Marginal likelihood estimation was implemented via path and stepping-stone modeling and Bayes factors were used to determine which of the three models best fit the data following Pečnerová et al. [67] (Additional file 1: Table S7). Convergence was determined and demographic reconstructions visualised in Tracer v1.7.1 [68]. The substitution rate was estimated from the data, where median ages were used as tipdates, with priors listed in Additional file 1: Table S5.

We tested four demographic models using Approximate Bayesian Computation (ABC) of simulated data generated using fastsimcoal v2.7.09 [69]. The models were as follows: (1) constant population size through time, (2) LGM bottleneck, (3) Eemian bottleneck, and (4) LGM and Eemian bottleneck. We accounted for population structure by having 13 distinct populations representing different temporal points of the five distinct clades (Additional file 1: Table S8). The mrca for each clade (as estimated above) were included. All models were specified using template (.tpl) and estimation (.est) files. The template files included variables that were then listed as priors in the estimation file (i.e. the timing and extent of bottlenecks). We estimated the timing of the bottlenecks using a log uniform prior for the LGM (30–20 kya) and the Eemian interglacial (130–115 kya) and the relative extent of the bottlenecks (0.2–0.6, note that this was set to 1.0 for the constant model). 500,000 simulations were run for each model using fastsimcoal v2.7.09 [69]. Summary statistics (including: number of segregating sites per population, mean, standard deviation and total number of segregating sites, nucleotide diversity (*π*) per population, mean and standard deviation *π*, and pairwise Fst for all comparisons between populations; n = 107) were computed using arlsumstat v3.5 from the Arlequin software package [70]. The ‘abc’ package in R [71] was used to determine the best fit model using a rejection model and a tolerance of 0.05.

### Nuclear genome analyses

Genome-wide heterozygosity and runs of homozygosity (ROH) were estimated using a development version of GenErode. We investigated a range of parameters for ROH, varying the number of windows (homozgy-window-snp) and heterozygous sites per window (homozgy-window-het): (1) homozgy-window-snp 100, homozgy-window-het 5; (2) homozgy-window-snp 250, homozgy-window-het 3; (3) homozgy-window-snp 100, homozgy-window-het 1. The following parameters remained constant through the three analyses above were: if at least 5% of all windows that included a given SNP were defined as homozygous, the SNP was defined as being in a ROH (homozyg-window-threshold 0.05); ROH segments had less than ≥ 25 SNPs (homozyg-snp 25), covered ≥ 100 kb (homozyg-kb 100) and had less than 15 missing sites (homozyg-window-missing 15); the minimum SNP density was one per 50 kb homozyg-density 50) and the maximum distance between two neighbouring SNPs was ≤ 1000 kb (homozyg-gap 1000); the number of heterozygous sites was set to 750 to prevent sequencing errors cutting ROHs (homozyg-het 750)*.*

Lastly, we investigated the demographic history using the Pairwise Sequential Markovian Coalescent (PSMC) approach [21]. This method reconstructs the effective population size (*N*_*e*_) over time by inferring the time to the most recent common ancestor (mrca) between two alleles on each chromosome based on the density of heterozygous sites. More ancient coalescent events are reflected in short regions of high heterozygosity, and more recent coalescent events reflected in long regions of low heterozygosity. The rate of coalescence is then inversely proportional to *N*_*e*_. Consensus sequences for all the scaffolds were generated with SAMtools mpileup v1.8 and the vcfutils.pl command ‘vcf2fq’. In this step we excluded repetitive regions, CpG sites, and short (< 10kbp) scaffolds, and filtered for mapping quality and depth. PSMC was run using the default parameters (-N25 -t15 -r5 -d -p "4 + 25*2 + 4 + 6"). The analysis was scaled using a generation time of two generations per year [23] and we tested three mutation rates estimated for the mouse (*Mus musculus*): minimum—4.6 × 10^–9^, average—5.4 × 10^–9^, and maximum—6.4 × 10^–9^ substitutions per site per generation [22] (Additional file 2: Fig S1).

## Supplementary Information

**Additional file 1: Table S1.** Modern Nuclear Genome sample information. **Table S2.** Ancient Mitogenome Sample Information. **Table S3.** Modern Mitogenome Sample Information. **Table S4.** Divergence times for each node in the phylogenetic tree estimated using BEAST. **Table S5.** The priors used to specify tip-date information for each ancient sample in the phylogenetic analysis. **Table S6.** Demographic Modelling using Approximate Bayesian Computation. **Table S7.** Marginal Likelihood Estimation for demographic models: constant size, skyline, skyride. **Table S8.** Information used to generate the simulated data for the ABC analysis.

**Additional file 2:** Supplementary materials and methods. Information regarding supplemental methods. **Figure S1.** PSMC plot for *Dicrostonyx torquatus* using three different mutation rates estimated for mouse (*Mus musculus*).

## Data Availability

Raw sequencing data for the de novo genome assembly and aligned BAM files for all mitogenome samples are available on ENA (Project number: PRJEB57187).

## References

[1] Brace S, Palkopoulou E, Dalen L, Lister AM, Miller R, Otte M (2012). Serial population extinctions in a small mammal indicate Late Pleistocene ecosystem instability. Proc Natl Acad Sci.

[2] Palkopoulou E, Baca M, Abramson NI, Sablin M, Socha P, Nadachowski A (2016). Synchronous genetic turnovers across Western Eurasia in Late Pleistocene collared lemmings. Glob Chang Biol.

[3] Campos PF, Willerslev E, Sher A, Orlando L, Axelsson E, Tikhonov A (2010). Ancient DNA analyses exclude humans as the driving force behind late Pleistocene musk ox (Ovibos moschatus) population dynamics. Proc Natl Acad Sci U S A.

[4] Lorenzen ED, Nogués-Bravo D, Orlando L, Weinstock J, Binladen J, Marske KA (2011). Species-specific responses of Late Quaternary megafauna to climate and humans. Nature.

[5] Stewart JR, Lister AM, Barnes I, Dalén L (2010). Refugia revisited: individualistic responses of species in space and time. Proc Biol Sci.

[6] Hewitt G (2000). The genetic legacy of the Quaternary ice ages. Nature.

[7] Metcalf JL, Prost S, Nogués-Bravo D, DeChaine EG, Anderson C, Batra P (2014). Integrating multiple lines of evidence into historical biogeography hypothesis testing: a Bison bison case study. Proc Biol Sci.

[8] Agadzhanyan AK. The history of collared lemmings in the Pleistocene. In: Beringia in the Cenozoic era; the Bering land bridge and its role in the history of holarctic floras and faunas in the late Cenozoic. Symposium. pascal-francis.inist.fr; 1984. p. 379–88.

[9] Kowalski K. Lemmings [Mammalia, Rodentia] as indicators of temperature and humidity in the European Quaternary. Acta Zool Cracov. 1995;38:85–94.

[10] Markova AK, Smirnov NG, Kozharinov AV, Kazantseva NE, Simakova AN, Kitaev LM. Late Pleistocene distribution and diversity of mammals in Northern Eurasia (PALEOFAUNA database). Paleontologia i Evolucio. 1995;28–29:5–143.

[11] Stewart JR, Van Kolfschoten M, Markova A, Musil R. The mammalian faunas of Europe during oxygen isotope stage three. In: van Andel TH, Davies SW, editors. Neanderthals and modern humans in the European landscape during the Last Glaciation, 60,000 to 20,000 years ago: archaeological results of the Stage 3 Project. McDonald Institute Monograph Series; 2003. p. 103–29.

[12] Ponomarev D, Puzachenko A (2015). Evolution of occlusal shape of the first and second upper molars of Middle-Late Pleistocene collared lemmings (Dicrostonyx, Arvicolinae, Rodentia) in northeast European Russia. Boreas.

[13] Prost S, Smirnov N, Fedorov VB, Sommer RS, Stiller M, Nagel D (2010). Influence of climate warming on arctic mammals? new insights from ancient DNA studies of the collared lemming Dicrostonyx torquatus. PLoS ONE.

[14] Fedorov VB, Trucchi E, Goropashnaya AV, Waltari E, Whidden SE, Stenseth NC (2020). Impact of past climate warming on genomic diversity and demographic history of collared lemmings across the Eurasian Arctic. Proc Natl Acad Sci U S A.

[15] Svensson A, Andersen KK, Bigler M, Clausen HB, Dahl-Jensen D, Davies SM (2006). The Greenland Ice Core Chronology 2005, 15–42 ka. Part 2: comparison to other records. Quat Sci Rev.

[16] Lagerholm VK, Sandoval-Castellanos E, Ehrich D, Abramson NI, Nadachowski A, Kalthoff DC (2014). On the origin of the Norwegian lemming. Mol Ecol.

[17] Lagerholm VK, Norén K, Ehrich D, Ims RA, Killengreen ST, Abramson NI (2017). Run to the hills: gene flow among mountain areas leads to low genetic differentiation in the Norwegian lemming. Biol J Linn Soc Lond.

[18] Smith S, Sandoval-Castellanos E, Lagerholm VK, Napierala H, Sablin M, Von Seth J (2017). Nonreceding hare lines: genetic continuity since the Late Pleistocene in European mountain hares (Lepus timidus). Biol J Linn Soc Lond.

[19] Larsson P, von Seth J, Hagen IJ, Götherström A, Androsov S, Germonpré M (2019). Consequences of past climate change and recent human persecution on mitogenomic diversity in the arctic fox. Philos Trans R Soc Lond B Biol Sci.

[20] Murton JB, Opel T, Toms P, Blinov A, Fuchs M, Wood J, et al. A multimethod dating study of ancient permafrost, Batagay megaslump, east Siberia. Quat Res. 2021;105:1–22.

[21] Li H, Durbin R (2011). Inference of human population history from individual whole-genome sequences. Nature.

[22] Uchimura A, Higuchi M, Minakuchi Y, Ohno M, Toyoda A, Fujiyama A (2015). Germline mutation rates and the long-term phenotypic effects of mutation accumulation in wild-type laboratory mice and mutator mice. Genome Res.

[23] Ehrich D, Jorde PE (2005). High genetic variability despite high-amplitude population cycles in lemmings. J Mammal.

[24] Funder S, Hjort C, Landvik JY, Nam S-I, Reeh N, Stein R (1998). History of a stable ice margin—East Greenland during the middle and Upper Pleistocene. Quat Sci Rev.

[25] Fedorov VB, Goropashnaya AV (1999). The importance of ice ages in diversification of arctic collared lemmings (Dicrostonyx): evidence from the mitochondrial cytochrome b region. Hereditas.

[26] Palkopoulou E, Dalén L, Lister AM, Vartanyan S, Sablin M, Sher A (2013). Holarctic genetic structure and range dynamics in the woolly mammoth. Pro R Soc B Biol Sci.

[27] Lord E, Dussex N, Kierczak M, Díez-del-Molino D, Ryder OA, Stanton DWG (2020). Pre-extinction demographic stability and genomic signatures of adaptation in the woolly rhinoceros. Curr Biol.

[28] Harington CR (2011). Pleistocene vertebrates of the Yukon Territory. Quat Sci Rev.

[29] Fedorov VB, Trucchi E, Goropashnaya AV, Chr SN (2022). Conflicting nuclear and mitogenome phylogenies reveal ancient mitochondrial replacement between two North American species of collared lemmings (Dicrostonyx groenlandicus, D. hudsonius). Mol Phylogenet Evol..

[30] Markova AK, Puzachenko AY, van Kolfschoten T, van der Plicht J, Ponomarev DV (2013). New data on changes in the European distribution of the mammoth and the woolly rhinoceros during the second half of the Late Pleistocene and the early Holocene. Quat Int.

[31] Heller R, Chikhi L, Siegismund HR (2013). The confounding effect of population structure on Bayesian skyline plot inferences of demographic history. PLoS ONE.

[32] Mather N, Traves SM, Ho SYW (2020). A practical introduction to sequentially Markovian coalescent methods for estimating demographic history from genomic data. Ecol Evol.

[33] Mazet O, Rodríguez W, Grusea S, Boitard S, Chikhi L (2016). On the importance of being structured: instantaneous coalescence rates and human evolution—lessons for ancestral population size inference?. Heredity.

[34] Palkopoulou E, Mallick S, Skoglund P, Enk J, Rohland N, Li H (2015). Complete genomes reveal signatures of demographic and genetic declines in the woolly mammoth. Curr Biol.

[35] Binney HA, Willis KJ, Edwards ME, Bhagwat SA, Anderson PM, Andreev AA (2009). The distribution of late-Quaternary woody taxa in northern Eurasia: evidence from a new macrofossil database. Quat Sci Rev.

[36] Tarasov PE, Andreev AA, Anderson PM, Lozhkin AV, Leipe C, Haltia E (2013). A pollen-based biome reconstruction over the last 3.562 million years in the Far East Russian Arctic—new insights into climate—vegetation relationships at the regional scale. Clim Past..

[37] Bigelow NH, Brubaker LB, Edwards ME, Harrison SP, Prentice IC, Anderson PM, et al. Climate change and Arctic ecosystems: 1. Vegetation changes north of 55°N between the last glacial maximum, mid-Holocene, and present. J Geophys Res. 2003;108:8170.

[38] Loog L, Thalmann O, Sinding M-HS, Schuenemann VJ, Perri A, Germonpré M (2020). Ancient DNA suggests modern wolves trace their origin to a Late Pleistocene expansion from Beringia. Mol Ecol.

[39] Sikora M, Pitulko VV, Sousa VC, Allentoft ME, Vinner L, Rasmussen S (2019). The population history of northeastern Siberia since the Pleistocene. Nature.

[40] Boessenkool S, Hanghøj K, Nistelberger HM, Der Sarkissian C, Gondek AT, Orlando L (2017). Combining bleach and mild predigestion improves ancient DNA recovery from bones. Mol Ecol Resour.

[41] Damgaard PB, Margaryan A, Schroeder H, Orlando L, Willerslev E, Allentoft ME (2015). Improving access to endogenous DNA in ancient bones and teeth. Sci Rep.

[42] Knapp M, Clarke AC, Horsburgh KA, Matisoo-Smith EA (2012). Setting the stage–building and working in an ancient DNA laboratory. Ann Anatomy-Anatomischer Anzeiger.

[43] Yang DY, Eng B, Waye JS, Dudar JC, Saunders SR. Technical note: improved DNA extraction from ancient bones using silica-based spin columns. 1998;543 December 1997:539–43. 10.1002/(SICI)1096-8644(199804)105:4<539::AID-AJPA10>3.0.CO;2-1 9584894

[44] Ersmark E, Orlando L, Sandoval-Castellanos E, Barnes I, Barnett R, Stuart A (2015). Population demography and genetic diversity in the Pleistocene Cave Lion. Open Quat.

[45] Meyer M, Kircher M (2010). Illumina sequencing library preparation for highly multiplexed target capture and sequencing. Cold Spring Harb Protoc..

[46] Baca M, Doan K, Sobczyk M, Stankovic A, Węgleński P (2012). Ancient DNA reveals kinship burial patterns of a pre-Columbian Andean community. BMC Genet.

[47] Baca M, Popović D, Lemanik A, Baca K, Horáček I, Nadachowski A (2019). Highly divergent lineage of narrow-headed vole from the Late Pleistocene Europe. Sci Rep.

[48] Horn S, Shapiro B, Hofreiter M (2012). Target enrichment via DNA hybridization capture. Ancient DNA: methods and protocols.

[49] Maricic T, Whitten M, Pääbo S (2010). Multiplexed DNA sequence capture of mitochondrial genomes using PCR products. PLoS ONE.

[50] Li R, Zhu H, Ruan J, Qian W, Fang X, Shi Z (2010). De novo assembly of human genomes with massively parallel short read sequencing. Genome Res.

[51] Butler J, MacCallum I, Kleber M, Shlyakhter IA, Belmonte MK, Lander ES (2008). ALLPATHS: de novo assembly of whole-genome shotgun microreads. Genome Res.

[52] Simpson JT, Wong K, Jackman SD, Schein JE, Jones SJM, Birol I (2009). ABySS: a parallel assembler for short read sequence data. Genome Res.

[53] Seppey M, Manni M, Zdobnov EM, Kollmar M (2019). BUSCO: assessing genome assembly and annotation completeness. Gene prediction: methods and protocols.

[54] Kutschera VE, Kierczak M, van der Valk T, von Seth J, Dussex N, Lord E (2022). GenErode: a bioinformatics pipeline to investigate genome erosion in endangered and extinct species. BMC Bioinform.

[55] Kearse M, Moir R, Wilson A, Stones-Havas S, Cheung M, Sturrock S (2012). Geneious Basic: an integrated and extendable desktop software platform for the organization and analysis of sequence data. Bioinformatics.

[56] Hahn C, Bachmann L, Chevreux B (2013). Reconstructing mitochondrial genomes directly from genomic next-generation sequencing reads—a baiting and iterative mapping approach. Nucleic Acids Res.

[57] Schubert M, Lindgreen S, Orlando L (2016). AdapterRemoval v2: rapid adapter trimming, identification, and read merging. BMC Res Notes.

[58] Li H, Durbin R (2010). Fast and accurate long-read alignment with Burrows-Wheeler transform. Bioinformatics.

[59] Li H, Handsaker B, Wysoker A, Fennell T, Ruan J, Homer N (2009). The sequence alignment/map (SAM) format and SAMtools. Bioinformatics.

[60] Milne I, Stephen G, Bayer M, Cock PJA, Pritchard L, Cardle L (2013). Using Tablet for visual exploration of second-generation sequencing data. Brief Bioinform.

[61] Bolger AM, Lohse M, Usadel B (2014). Trimmomatic: a flexible trimmer for Illumina sequence data. Bioinformatics.

[62] Fedorov VB, Goropashnaya AV (2016). Complete mitochondrial genomes of the North American collared lemmings Dicrostonyx groenlandicus Traill, 1823 and Dicrostonyx hudsonius Pallas, 1778 (Rodentia: Arvicolinae). Mitochondrial DNA B Resour.

[63] Edgar RC (2004). MUSCLE: a multiple sequence alignment method with reduced time and space complexity. BMC Bioinform.

[64] Darriba D, Taboada GL, Doallo R, Posada D (2012). jModelTest 2: more models, new heuristics and parallel computing. Nat Methods.

[65] Rambaut A. FigTree, a graphical viewer of phylogenetic trees. See http://tree.bio.ed.ac.uk/software/figtree. 2007.

[66] Suchard MA, Lemey P, Baele G, Ayres DL, Drummond AJ, Rambaut A. Bayesian phylogenetic and phylodynamic data integration using BEAST 1.10. Virus Evol. 2018;4:vey016. 10.1093/ve/vey016 PMC6007674 29942656

[67] Pečnerová P, Palkopoulou E, Wheat CW, Skoglund P, Vartanyan S, Tikhonov A (2017). Mitogenome evolution in the last surviving woolly mammoth population reveals neutral and functional consequences of small population size. Evol Lett.

[68] Rambaut A, Drummond AJ, Xie D, Baele G, Suchard MA (2018). Posterior summarization in Bayesian Phylogenetics using tracer 1.7. Syst Biol..

[69] Excofffier L, Marchi N, Marques DA, Matthey-Doret R, Gouy A, Sousa VC (2021). fastsimcoal2: demographic inference under complex evolutionary scenarios. Bioinformatics.

[70] Excoffier L, Lischer HEL (2010). Arlequin suite ver 3.5: a new series of programs to perform population genetics analyses under Linux and Windows. Mol Ecol Resour..

[71] Csilléry K, François O, Blum MGB (2012). abc: An R package for approximate Bayesian computation (ABC). Methods Ecol Evol.

