# Supplementary material for: Population dynamics and demographic history of Eurasian collared lemmings
Source: BMC Ecol Evol. 2022 Nov 3;22:126. doi: 10.1186/s12862-022-02081-y (PMC9632076; doi:10.1186/s12862-022-02081-y)
Supplement: Supplementary file 2 — Additional file 2: Supplementary materials and methods. Information regarding supplemental methods. Figure S1. PSMC plot for Dicrostonyx torquatus using three different mutation rates estimated for mouse (Mus musculus). [file 12862_2022_2081_MOESM2_ESM.docx]

**Additional Information:**

Lord et al. **Population dynamics and demographic history of Eurasian collared lemmings**

**Materials and Methods**

*Sample collection*

For this study we collected a total of 127 samples (mandibles) of *Dicrostonyx torquatus* from 13 archaeological and paleontological sites. The DNA extractions and all pre-PCR work on the samples were performed in dedicated ancient DNA laboratories at the Swedish Museum of Natural History (NRM) and Centre for Palaeogenetics (CPG) in Stockholm (Sweden), and the Laboratory of Paleogenetics and Conservation Genetics, Centre of New Technologies, University of Warsaw (LPCG) in Warsaw (Poland). All the procedures were performed with sterilized equipment and to minimize the risk of contamination from exogenous sources and between the samples [[1]](https://paperpile.com/c/4fudll/AdcfJ). Negative controls were included during DNA extraction, library preparation and indexing PCR phases. Similarly, all the post-PCR work was done in a second dedicated laboratory and both these laboratories are physically separated from each other.

For the *de-novo* genome assembly, tissue was collected from a wild-caught female *Dicrostonyx torquatus* from Eastern Siberia (http://arctos.database.museum/guid/UAM:Mamm:84102).

*DNA Extraction, Library preparation and Sequencing*

For the EL and MAM labelled samples, we performed a modified bleach and pre-digestion protocol [[2, 3]](https://paperpile.com/c/4fudll/8YnmE+fbLHV) on mandible fragments to reduce surface contamination and increase endogenous DNA content. Specifically, the samples were wrapped in aluminium foil and reduced to fragments (not powder) and approximately 5-187 mg of the bone fragments were transferred to a 1.5 ml Eppendorf screw cap tube and 500 μl of 0.5% bleach was added.

The samples were incubated at 25°C for 10 minutes under rotation and centrifuged for 2 minutes at 2,300 rpm. The bleach was removed and the samples were washed 3 times with 500 μl distilled water. For the pre-digestion, we added 700 μl of extraction buffer (comprised of 630 μl of 0.5 M, pH 8 EDTA,70 μl of 1M UREA and 15 μl of 10 µg/µL Proteinase K) to the bone fragments and incubated them at 55°C for 30 minutes, under rotation. DNA extraction was performed following Protocol C [[4]](https://paperpile.com/c/4fudll/eosIw) with modifications as per Ersmark *et al.* [*[5]*](https://paperpile.com/c/4fudll/az67e/?noauthor=1).

Double stranded Illumina DNA libraries were constructed following Meyer and Kircher [[6]](https://paperpile.com/c/4fudll/2CDpI) from a starting volume of 20 μl of DNA extract, with an additional step of incubation with uracil-specific excision reagent (USER enzyme) (New England Biolabs) [[7]](https://paperpile.com/c/4fudll/ywizD), to remove DNA damage characteristic of ancient samples. Blunt end repair was performed in a 40 ul reaction: 1× buffer Tango, 100 μM of each dNTP, 1 mM ATP, 25 U T4 polynucleotide kinase (Thermo Scientific) and 3U USER enzyme (New England Biolabs) and incubated samples were incubated for 3 hours at 37°C, followed by the addition of 1 μl T4 DNA polymerase (Thermo Scientific) and incubation at 25°C for 15 min and 12°C for 5 min. Reactions were then purified using MinElute PCR columns (Qiagen). Adapter ligation step was performed by adding to 20 µl of purified DNA to the following adapter ligation mix: 10 μM adapter mix, 1X T4 DNA ligase buffer, 5% PEG-4000, 0.125 U T4 DNA Ligase. The samples were then incubated for 30 minutes to 22°C and purified using MinElute PCR columns (Qiagen). Finally, we performed the adapter fill in adding to 20 µl of purified DNA the following adapter fill in master mix: 1X Thermopol reaction buffer, 250 µM dNTPs, 0.3 U Bst polymerase. The samples were incubated in the thermocycler for 20 minutes at 37°C followed by heat-inactivation at 80°C for 20 minutes.

The DNA libraries were amplified using a modified version of protocol in Meyer and Kircher [[6]](https://paperpile.com/c/4fudll/2CDpI) using 3μl of adapter-ligated library as template, with the following final concentrations: 1x AccuPrime reaction mix, 0.3 μM P7 indexing primer, 0.3 μM P7 indexing primer, 7 U AccuPrime Pfx (Thermo Scientific). Dual unique indexes (6bp) were added to each sample, and amplified using the following conditions: 95°C for 2 minutes and 95°C for 15 seconds, 60°C for 30 seconds, and 68°C for 30 seconds. The number of PCR cycles varied, from 12 to 18 cycles, depending on the concentration of DNA in the sample. We visualised the presence of DNA in samples using gel electrophoresis, using 5 µl PCR products on a 1.5% agarose gel prepared with fluorescent GelGreen (Biotium Inc.). The gel was run in 1 x TAE buffer and inspected under UV light.

To purify and simultaneously conduct a size selection of the post-amplified libraries, we used Agencourt AMPure XP beads (Beckman Coulter). Fragments more than 500bp (likely contaminant sequences) and less than 100bp (likely adapter dimers) were removed. Samples were pooled in equimolar ratio and quantified using a Bioanalyzer 2100 (Agilent). Pooled libraries were sequenced on either a 2x50bp setup on an Illumina SPrime or Illumina NovaSeq S4 2x100bp setup at Sci*Life*Lab, Stockholm.

The L-labeled specimens were processed in ancient DNA facilities at LPCG. We used DNA extracts obtained previously using a modified phenol-chloroform method [[8]](https://paperpile.com/c/4fudll/S2lh2). Double-indexed double-stranded sequencing libraries were built from 20 µl of DNA extracts following the protocol by Meyer and Kircher [[6]](https://paperpile.com/c/4fudll/2CDpI), with minor modifications [[9]](https://paperpile.com/c/4fudll/XKMMj). Indexing PCR was performed using AmpliTaq Gold 360 Master Mix (Applied Biosystems) with 19 amplification cycles. PCR products from three independent amplifications were pooled, purified and eluted with 40 µl of EBT buffer. In-solution hybridization capture approach was used to enrich libraries in mitochondrial DNA. Hybridization bait was produced using the mtDNA of modern specimens of several species of small mammals: common vole (*Microtus arvalis*), field vole (*Microtus agrestis*), root vole (*Microtus oeconomus*), bank vole (*Clethrionomys glareolus*) and narrow-headed vole (*Lasiopodomys gregalis*). DNA was extracted from tissue fragments using the DNeasy Blood & Tissue Kit (Qiagen). Complete mitochondrial genomes were amplified as several overlapping fragments. PCR products were sonicated to an average fragment length of 200 bp using Covaris S220 and transformed into bait following Maricic *et al.* [*[10]*](https://paperpile.com/c/4fudll/Rsmj5).

Two rounds of hybridization were performed following Horn [[11]](https://paperpile.com/c/4fudll/GHlRN). We used hybridization temperature of 55°C to account for the high divergence between mtDNA of species used for bait construction and the target species. Each round of hybridization was carried out for 22-24 h. After each round, for each library pool, three parallel post-capture PCRs were performed using Herculase II Fusion Polymerase (Agilent) at 15 cycles. Products amplified after the second round were purified, quantified, pooled in equimolar ratios and paired-end sequenced on Illumina NextSeq platform (2x150bp) in MidOutput mode. In order to estimate endogenous DNA, uncaptured libraries were also shotgun sequenced on an Illumina Nextseq 550 in a 2x150bp, mid-output, paired-end setup.

**References**

[1. Knapp M, Clarke AC, Horsburgh KA, Matisoo-Smith EA. Setting the stage–building and working in an ancient DNA laboratory. Annals of Anatomy-Anatomischer Anzeiger. 2012;194:3–6.](http://paperpile.com/b/4fudll/AdcfJ)

[2. Boessenkool S, Hanghøj K, Nistelberger HM, Der Sarkissian C, Gondek AT, Orlando L, et al. Combining bleach and mild predigestion improves ancient DNA recovery from bones. Mol Ecol Resour. 2017;17:742–51.](http://paperpile.com/b/4fudll/8YnmE)

[3. Damgaard PB, Margaryan A, Schroeder H, Orlando L, Willerslev E, Allentoft ME. Improving access to endogenous DNA in ancient bones and teeth. Sci Rep. 2015;5:11184.](http://paperpile.com/b/4fudll/fbLHV)

[4. Yang DY, Eng B, Waye JS, Dudar JC, Saunders SR. Technical Note : Improved DNA Extraction From Ancient Bones Using Silica-Based Spin Columns. 1998;543 December 1997:539–43.](http://paperpile.com/b/4fudll/eosIw)

[5. Ersmark E, Orlando L, Sandoval-Castellanos E, Barnes I, Barnett R, Stuart A, et al. Population Demography and Genetic Diversity in the Pleistocene Cave Lion. OPEN QUAT. 2015;1:4.](http://paperpile.com/b/4fudll/az67e)

[6. Meyer M, Kircher M. Illumina sequencing library preparation for highly multiplexed target capture and sequencing. Cold Spring Harb Protoc. 2010;2010:db. prot5448.](http://paperpile.com/b/4fudll/2CDpI)

[7. Briggs AW, Stenzel U, Meyer M, Krause J, Kircher M, Pääbo S. Removal of deaminated cytosines and detection of in vivo methylation in ancient DNA. Nucleic Acids Res. 2009;38:e87.](http://paperpile.com/b/4fudll/ywizD)

[8. Palkopoulou E, Baca M, Abramson NI, Sablin M, Socha P, Nadachowski A, et al. Synchronous genetic turnovers across Western Eurasia in Late Pleistocene collared lemmings. Glob Chang Biol. 2016;22:1710–21.](http://paperpile.com/b/4fudll/S2lh2)

[9. Baca M, Popović D, Lemanik A, Baca K, Horáček I, Nadachowski A. Highly divergent lineage of narrow-headed vole from the Late Pleistocene Europe. Sci Rep. 2019;9:17799.](http://paperpile.com/b/4fudll/XKMMj)

[10. Maricic T, Whitten M, Pääbo S. Multiplexed DNA sequence capture of mitochondrial genomes using PCR products. PLoS One. 2010;5:e14004.](http://paperpile.com/b/4fudll/Rsmj5)

[11. Horn S. Target Enrichment via DNA Hybridization Capture. In: Shapiro B, Hofreiter M, editors. Ancient DNA: Methods and Protocols. Totowa, NJ: Humana Press; 2012. p. 177–88.](http://paperpile.com/b/4fudll/GHlRN)


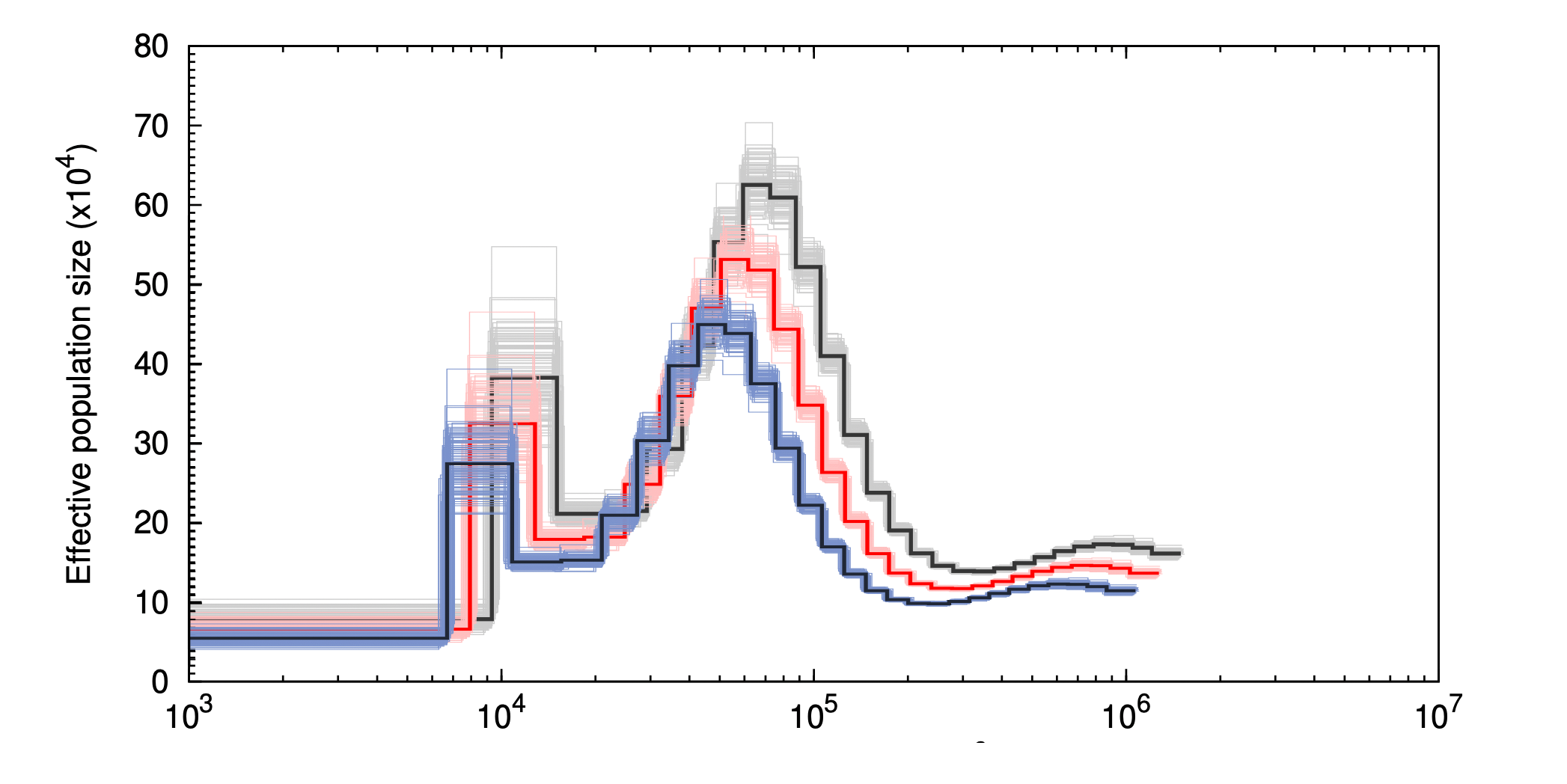


**Figure S1:** PSMC plot for *Dicrostonyx torquatus* using three different mutation rates estimated for mouse (*Mus musculus*) obtained from Uchimura *et al.* [*[25]*](https://paperpile.com/c/4fudll/OtP4g/?noauthor=1). The y-axis shows effective population size and the x-axis shows time in years from 10^3^ to 10^7^ years ago. The black line shows the minimum mutation rate (4.6×10^-9^ substitutions per site per generation), the red line the average mutation rate (5.4×10^-9^ substitutions per site per generation) and the blue line the maximum mutation rate (6.4×10^-9^ substitutions per site per generation). Lighter colours represent the 95% confidence interval for each of the curves estimated by PSMC. All curves used a generation time of two generations per year.
